# An Exploratory Investigation of Government Air Monitoring Data after Hurricane Harvey

**DOI:** 10.3390/ijerph19095559

**Published:** 2022-05-03

**Authors:** Kwanita Adair, Shelly Miller, Margot Gage Witvliet

**Affiliations:** 1 Department of Sociology, Social Work and Criminal Justice, Lamar University, Beaumont, TX 77705, USA; kadair1@lamar.edu; 2 Department of Mechanical Engineering, University of Colorado Boulder, Boulder, CO 80309, USA; shelly.miller@colorado.edu

**Keywords:** air pollution, air quality, environment and health, climate change, environmental justice

## Abstract

Southeast Texas is home to some of the largest refineries in the United States. During Hurricane Harvey, emergency shutdowns took place. In this exploratory investigation, we examine how government air monitors performed in measuring air quality in Beaumont, Texas during and in the months following Hurricane Harvey. Texas Commission on Environmental Quality (TCEQ) data from two active air monitors in Beaumont, Texas were analyzed during the year 2017–2018. Concentrations of sulfur dioxide (SO_2_), nitric oxide (NO), oxides of nitrogen (NOx), ozone, benzene, and hydrogen sulfide (H_2_S) were investigated. The number of hours and days no data were reported by air monitors were also investigated. Yearly maximum values (MAX, all in parts per billion (ppb)) in 2017 for SO_2_, NO, and NOx (53.7, 113.4, 134, respectively) and their respective standard deviations (SD: 1.3, 3.4, and 14) were higher as compared to 2018 (MAX, all in ppb and (SD) = 40.9, (1.4); 103.9, (3.3); 123.8, (14), respectively). The data capture rate for these chemicals were between 88 and 97% in both years. During the months following Hurricane Harvey (August–December 2017) there was an increase in most maximum values. The yearly averages for H_2_S were 0.68 ppb (SD 1.02) in 2017 and 0.53 ppb (SD 1.07) in 2018. Missing days were observed for both the H_2_S and NOx air monitors, with the highest number observed in 2017 (213 missing days) for the air monitor measuring H_2_S. We identified that residents of Beaumont, Texas are exposed daily to low-level concentrations of air pollutants. H_2_S is released each day at a level high enough to be smelled. Data capture rates for air monitors are not always above 90%. Improved air quality data and disaster preparations are needed in Beaumont, Texas.

## 1. Introduction

Air pollution is a worldwide public health concern [1]. Air pollution increases the chances of respiratory illnesses [2,3,4]. Children and the elderly are the most vulnerable groups for illness attributed to air pollution [5]. A link between the increase in air pollutants and deaths related to the heart and lungs is evident [6]. The World Health Organization estimates that 7 million people die from air pollution each year [7].

Prior studies conclude that those living near air-polluting petroleum refineries experience poorer health [8,9,10]. These people living near refineries also have a moderately higher risk of dying from lung cancer [11]. Living near environmental hazards is associated with pregnancy complications, childhood cancer, cardiovascular illness, and diabetes [12]. Irritation of the eyes, headaches, and dizziness are also reported health complaints when living in proximity to a refinery [13]. Natural disasters such as flooding, and hurricanes can make living in proximity to industry even worse [14]. Air pollutants released after a flood can damage the respiratory system. Chemicals from spills can contaminate water sources and soil, and this has the potential to negatively impact population health for years after a disaster [14].

In August 2017, Texas experienced Hurricane Harvey. According to the National Hurricane Center, Hurricane Harvey caused billions of U.S. dollars of damage, second to Hurricane Katrina [15,16]. Millions of pounds of air pollution were released during Hurricane Harvey [17]. Many Texas cities were impacted by Hurricane Harvey, but the city of Beaumont, Texas is unique in that the entire city is surrounded by industry, and when the flooding from Hurricane Harvey occurred, Beaumont, Texas —unlike the larger city of Houston—became a tiny island surrounded by polluting refineries. There was no way into or out of the city for days, except by boat or air.

A small city like Beaumont, Texas is easy to overlook on the map, but industry generates big business in this predominately African American city. The smell of air pollution is so common, residents refer to the pungent odor of rotten eggs as the “smell of money” [18]. Beaumont is the murder capital of Texas, with more crime per capita than Houston (the fourth largest city in the USA). The population of Beaumont, Texas is 118,296, with 48% being African American, 45% white, and 14% Latinx [19]. The average age of residents is 34 years [19]. Women make up 51% of the population, and the overall poverty rate is 19% [19]. Data from the Texas government show higher incidences of all cancers, including leukemia and respiratory cancer, in Beaumont compared to the rest of Texas [20]. Citizens of Beaumont not only die at a higher rate from cancer, but their chance of developing cancer is also higher than that of those in the rest of Texas. Age-adjusted incidence rates per 100,000 in Beaumont are 64.7 for respiratory cancer, 13.2 for leukemia, and 406.4 for all types of cancer. For comparison statewide, the incidence rates are 52.1, 12.9, and 391.8, respectively. Beaumont residents also have slightly higher levels of asthma than those in the rest of Texas [20].

Possible public health effects from Hurricane Harvey have yet to be investigated for the population of Beaumont, Texas. What we know is that between the cities of Beaumont, Corpus Christi, and Houston, Texas, it is estimated that about “40 petrochemical companies released more than 5.5 million pounds of chemicals as a result of the hurricane” [21]. In this exploratory investigation, we examine data from Texas Commission on Environmental Quality (TCEQ) air monitors pre- and post-Hurricane Harvey to investigate how the monitors performed in Beaumont, Texas during Hurricane Harvey.

**Figure 1:**
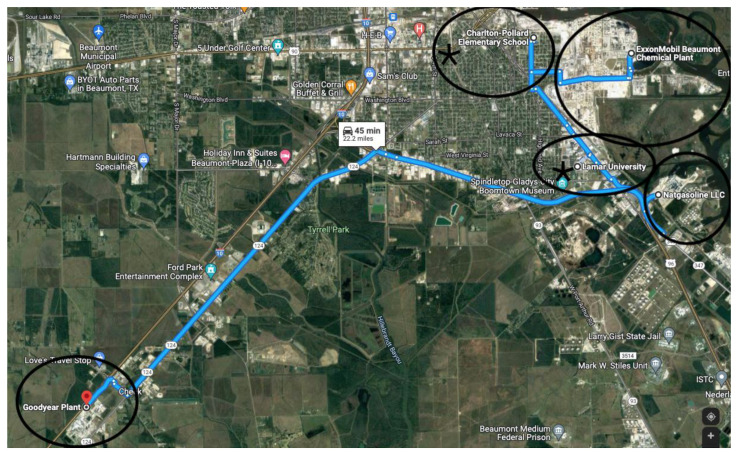
Map of Beaumont, Texas showing industries and location of two air monitors. The two stars on the map denote Beaumont Downtown (located within the Lamar University circle) and Beaumont Mary (located within the Charlton Pollard Elementary circle).

In Beaumont, Texas, we used TCEQ data to identify two active air monitors. Figure 1 shows the ExxonMobil Beaumont Chemical Plant, Natgasoline, LLC located in Beaumont, Texas USA Goodyear Plant, and its adjacent proximity to Charlton Pollard Elementary School and Lamar University (>3 miles) in Beaumont, Texas USA [22]. Not shown is the ExxonMobil Polyethylene Plant located 3.5 miles from Westbrook High school in Beaumont, Texas USA. In the entire city of Beaumont, we identified that there are only two active air monitors: (1) Beaumont Downtown and (2) Beaumont Mary. The Beaumont Downtown air monitor is located at 1086 Vermont Avenue, 3.2 miles away from one of the refineries, but is closest to the Port of Beaumont ship traffic [22]. The Port of Beaumont is the fifth largest port in the nation. Beaumont Mary is located at 598 Craig Street, 1.7 miles away from the refiner and nearest to the Charlton Pollard elementary school that is across the street from the refinery [22]. This exploratory investigation is novel because Beaumont, Texas is a city with one of the largest industries in the United States, yet with only two air monitors. The public is not aware of how the air monitors are performing, especially during a disaster.

## 2. Methods

To investigate how air monitors in the city performed during Hurricane Harvey, the data produced by the Texas Commission on Environmental Quality (TCEQ) air monitors were examined. TCEQ is an environmental agency that helps maintain public health and natural resources in Texas to uphold economic development [23]. TCEQ offers information on air, land, water, permit licenses, and reporting programs and facilities [23]. TCEQ data are freely available to the public online. We investigated the TCEQ air quality data that were available to the public for the years 2017–2018 [23].

The air pollutant data extracted from the TCEQ database for the Beaumont Downtown air monitoring site included: sulfur dioxide (SO_2_), nitric oxide (NO), nitrogen dioxide (NO_2_), oxides of nitrogen (NOx), ozone (O_3_), and benzene. The Beaumont Mary air monitoring site only measures hydrogen sulfide (H_2_S). In our investigation, air pollutant figures from the TCEQ database for both air monitors by year and month were reviewed. Next, we investigated the Beaumont, Mary air monitor by hour. Following this, we documented the number of days the air monitors were shut off. The aim of this approach was to obtain a snapshot overview of the air quality values during and after Hurricane Harvey.

## 3. Results

In Table 1, the yearly values (2017–2018) of chemicals, measured in parts per billion (ppb), are shown for the Beaumont Downtown. Data capture (CAP) is the percentage of valid data collected and MAX is the maximum value of the specified chemical reported by the air monitor. Yearly maximum values for SO_2_, NO, and NOx were higher during the 2017 Hurricane Harvey period (MAX, all in ppb = 53.7, standard deviation (SD) 1.3; 113.4, SD 3.4; 134.0, SD 14, CAP (in %) 95.8, 94.1, 95.6, respectively) as compared to 2018 (MAX, all in ppb = 40.9, SD 1.4; 103.9, SD 3.3; 123.8, SD 14; CAP (in %) 98.4, 97.9, 97.4, respectively). Yearly maximum values for NO_2_, O_3_, and benzene were higher in 2018. The calculated average values appear relatively similar from 2017 to 2018. In 2017, we identified 68 days of missing data for all chemicals and 22 days missing data for benzene.

**Figure 2:**
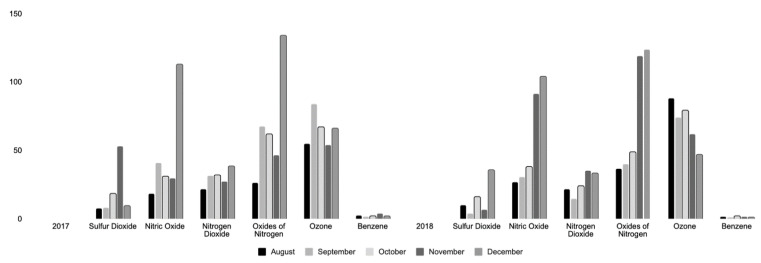
Beaumont Downtown Air Monitor Monthly Maximum Values in PPB For Air Pollutants, 2017-2018. Note: Figure 2 shows air monitoring data in parts per billion (PPB) for all pollutants from August to December in 2017 and 2018.

In Figure 2, monthly values of chemicals extracted from Beaumont Downtown for August–December 2017–2018 are shown. Sulfur dioxide shows a monthly increase in the maximum value from August 2017 to November 2017. In 2017, the maximum values for nitric oxide fluctuate from August to December. Nitrogen dioxide appears to show the highest maximum values in 2017, with the exception of August and November 2017. Oxides of nitrogen have the highest maximum values in December for both 2017 and 2018 (MAX, all in ppb = 134.0, SD 14; 123.8, SD 14; CAP (in %) = 97.4, 88.4, respectively).

In November, maximum values for sulfur dioxide and benzene were higher in 2017 as compared to 2018. Maximum values for ozone appear to increase from 2017 to 2018. The highest number of missing days for all pollutants (i.e., sulfur dioxide, nitric oxide, nitrogen dioxide, oxides of nitrogen, ozone, and benzene) were in August 2017 (7 days missing). The Data Capture (CAP) rate in August 2017 was 74–75%, which means a likely underestimation of values for the Hurricane Harvey period of 2017. Benzene appears to have the highest number of missing hours (87 h total) in August 2017.

In Table 2 the yearly values for H_2_S measured at Beaumont Mary for 2017–2018 are shown. A higher amount of H_2_S appears to have been released into the air in the year following the Hurricane Harvey period. However, given the number of missing days in 2017 as compared to 2018, the yearly value for H_2_S in 2017 appears to be an underestimation. TCEQ Beaumont Mary air monitor showed 213 missing days in 2017, and in 2018 this figured dropped to 24 missing days. The yearly average (AVG) for H_2_S concentration is 0.68 ppb, SD 1.02; 0.53 ppb, SD 1.07; CAP (in %) = 39.1, 92.5, respectively).

**Figure 3:**
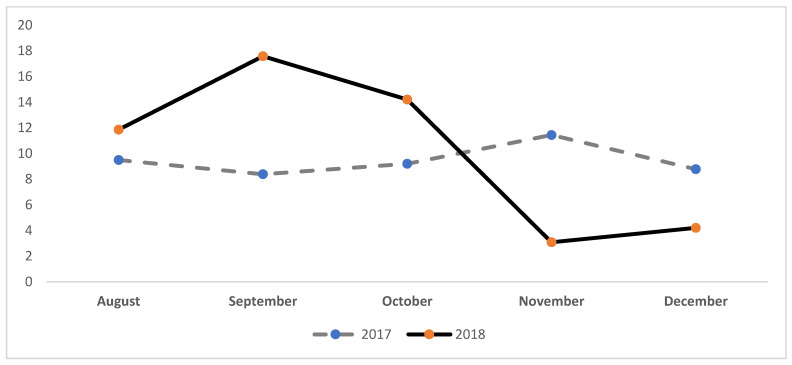
Beaumont Mary Air Monitor Monthly MAX Values, Hydrogen Sulfide 2017–2018. Note: Figure 3 shows air monitoring data in parts per billion (PPB) for H2S from August to December in 2017 and 2018.

In Figure 3 the monthly values for H_2_S are shown. The highest monthly maximum values for H_2_S from August–October 2017 are (MAX, all in ppb = 9.49, SD 1.06; 8.38, SD 0.95, 9.20, SD 1.09; CAP (in %) = 73, 90, 95.7, respectively) with an increase in concentration observed during the August–October 2018 period (MAX, all in ppb = 11.85, SD 0.93; 17.58, SD 1.36; 14.21, SD 1.10; CAP (in %) = 94.6, 95.4, 95, respectively). 

**Figure 4:**
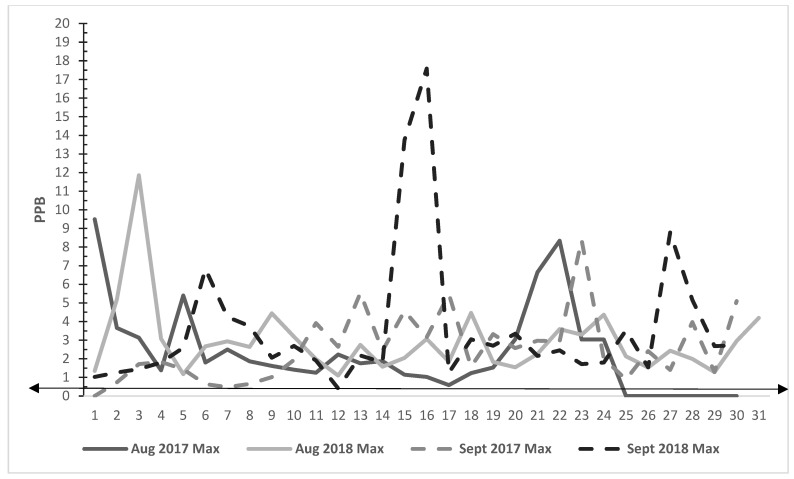
Beaumont Mary Air Monitor Hydrogen Sulfide MAX Values 30-Day Period, August 2017–2018 with Smell Line Arrow. Note: Figure 4 shows air monitoring data in parts per billion (PPB) for H_2_S for the month of August in 2017 and 2018. Missing data shown at end of the month of August in 2017. Smell line arrow signifies the level at which H2S can be smelled in the air.

In Figure 4, hourly values for H_2_S measured at Beaumont Mary for August and September 2017–2018 are shown. The highest hourly maximum value for H_2_S was in 2018 in August (MAX = 11.85 ppb; CAP = 73%) and increased in September to 17.58 ppb; CAP = 90%. The 24 h H_2_S average concentration was 0.14 ppb per day. In August and September 2018, measurements of H_2_S reached over 10 ppb on three different days. The maximum values for H_2_S in August and September exceeded 0.5 ppb in both 2017 and 2018. The minimum observed values exceeded 0.5 ppb at least once in August 2017 and in September 2018.

A total of 206 missing days are evident before Hurricane Harvey occurred. The yearly average data capture for the Beaumont Downtown monitor did not meet industry standards in 2017, at 39.1%. In 2018, the yearly data capture rate improved and was slightly above industry standard at 92.5%. For the Beaumont Mary monitor, data capture was below industry standard at 29.1% in 2017 and improved slightly above industry standard to 92.5% in 2018. Missing data during the Hurricane Harvey period show days missing from 25 August to 1 September 2017.

## 4. Conclusions

The aim of this exploratory investigation was not to provide a comprehensive overview of air quality in Beaumont, Texas. We examined how the air monitors in the city were performing during Hurricane Harvey. We identified that air quality in Beaumont, Texas appears to remain consistent from the Hurricane Harvey period (2017) and thereafter (2018). However, because of missing data during the Hurricane Harvey period and the reality of seasonal trends in pollution that are not accounted for in this investigation, it is possible that the air pollution levels for 2017 are an underestimation. A strength of this exploratory investigation is that data validated by TCEQ are used. The TCEQ air monitors use either federal reference or equivalent methods, and the data meet EPA quality assurance criteria and are used for regulatory purposes [23]. We are unaware of any data available from industrial continuous emissions monitors, typically installed in emissions stacks to document pollutant emission rates; these data are usually the preview of the industrial facility. As such, we utilized the best data available to the public.

We did not expect to identify that the data capture rate would be under 90% before the Hurricane Harvey period. The TCEQ explanation for missing data capture rate lists various reasons that data are rejected, including lost data and automatic calibration [23]. The low data capture rate before Hurricane Harvey suggests a need for more air monitors to be placed around the city, so that if one monitor is not performing optimally, there are multiple backup air monitors. The reason for missing data during the Hurricane Harvey period is that the TCEQ turned air monitors off to prevent them from being harmed during the storm [24]. During this time, government officials allowed industries to exceed permit mandates and temporarily release a higher amount of chemicals [24]. Industries were also allowed to self-report emissions to TCEQ. In situations of a disaster or in cities such as Beaumont, Texas with heavy industry, the use of unmanned aerial vehicles might be a useful solution [25].

Another issue that we identified is that neither Beaumont Downtown nor Beaumont Mary air monitors collect data for particulate matter (PM). PM was monitored 2010–2015 at Beaumont Mary and only in 2008 for Beaumont Downtown. PM_10_ or PM_2.5_ is distinguished by the size of the particles. PM is a mixture of liquid and non-liquid particles in the air [26]. PM is commonly created from pollutants emitted from power plants, industries, and cars [26]. Fine particles are dangerous because they can enter the lungs and bloodstream [26]. People with heart and lung disease die earlier when exposed to PM [26]. A meta-analysis found a link between increased PM and increased risks of lung cancer [27]. Exposure to PM_2.5_ over a short period is linked to increased chronic obstructive pulmonary disease hospitalizations and death [27]. 

The Beaumont Mary air monitor is located closest to the refinery, but measures only one chemical. This leaves little information to be gleaned on the status of air quality for those living closest to the refinery. The city of Beaumont, Texas should consider installing not only more air monitors around the city, but also air monitors that measure a multitude of air pollutants in closer proximity to the areas and neighborhoods where people live and where industry is located.

We investigated the maximum and average values of chemical concentrations. Exposure depends on both concentration and time spent breathing the contaminated air. Beaumont, Texas has not only long-term low-level concentrations of air pollutants, but also has an increased level of concentrations and potential exposure in the months following a disaster. We observed that residents of Beaumont, Texas can smell H_2_S in the air almost daily. This is noteworthy given that H_2_S can be problematic for people with respiratory issues, and exposure to low levels of H_2_S can trigger neurological problems [28]. Further investigation is needed to identify the extent to which H_2_S might be impacting the health of residents. What is known is that, in 2009, TCEQ removed Beaumont, Texas from the Air Pollutant Watch List for H_2_S [29]. Our observations of the air monitoring data measuring H_2_S suggest that an official review of the Beaumont Mary air monitor is warranted.

Considering the high concentration of industry in Beaumont, Texas, it is critical that the air-monitoring plan includes an objective discussion with relevant stakeholders on how to reduce the length of time air monitors are shut down, and an expansion of the collection of more robust air pollutant data. The city of Beaumont, Texas needs industry for economic reasons. Nevertheless, it is imperative that public health and economics work together. A city with this much industry, that also faces an increased regular risk of natural disasters, needs a more nuanced plan to protect public health from industry that pollutes.

## Figures and Tables

**Table 1:** Yearly values 2017–2018 chemicals measured at air monitor Beaumont Downtown.

	MAX	SH	MIN	AVG	SD	CAP (%)	Missing Hours	Missing Days
**Sulfur Dioxide** 2017 2018	53.7 40.9	52.9 38.7	−0.3 −0.4	0.5 0.5	1.3 1.4	95.8 98.4	179 138	8 0
**Nitric Oxide** 2017 2018	113.4 103.9	101.5 91.6	−0.7 −0.5	1.2 1.2	3.4 3.3	94.1 97.9	249 187	11 0
**Nitrogen Dioxide** 2017 2018	38.3 43.7	36.7 39.9	0.3 0	5.8 5.8	5.0 4.8	94.1 97.9	245 187	11 0
**Oxides of Nitrogen** 2017 2018	134.0 123.8	127.8 118.9	0.2 0	6.9 6.8	14 14	95.6 97.4	271 176	8 0
**Ozone** 2017 2018	88 96	84 94	−2 −4	24 23	14 14	95.6 97.4	192 153	8 3
**Benzene** 2017 2018	7.29 27.6	5.28 3.40	0 0	0.22 0.15	0.3 0.4	88.9 93.3	1103 1137	22 4

Note: MAX = Maximum Value, SH = Second Highest Value, MIN = Minimum Value, AVG=Average Value, SD = Standard Deviation and CAP % = Data Capture Rate.

**Table 2:** Hydrogen sulfide monthly and yearly values measured at air monitor Beaumont Mary August–December 2017–2018.

	MAX	SH	MIN	AVG	SD	CAP (%)	Missing Hours	Missing Days
2017	17.18	11.44	−0.33	**0.68**	1.02	39.1	195	213
2018	24.30	20.46	−0.40	**0.53**	1.07	92.5	583	3

Note: AVG value in the table is above EPA values of 0.14 ppb. (i.e., H_2_S EPA limit for children and elderly). Missing hours = denoted by TCEQ as calibration check. Missing days = a steady 24 h period of missing data. MAX = Maximum, SH= Second highest value, MIN = Minimum value, AVG = Average Value, SD = Standard Deviation, CAP = Data Capture. Values measured in parts per billion (ppb).

## Data Availability

TCEQ data available freely to the public online at https://www.tceq.texas.gov/ (accessed on 22 February 2022).
